# 

**Published:** 1846-04

**Authors:** 


### CONTENTS OF Nº XLII

OF THE

### British and Foreign Medical Review.

APRIL, 1846.

---

### Part First.—Analytical and Critical Reviews.

PAGE

ART. I.—Die Operative Chirurgie von JOHANN FRIEDRICH DIEFFENBACH. Erster Band . . . . . . . 287

The Operative Surgery of J. F. DIEFFENBACH. First Volume.

ART. II.—1. Second Report of the Commissioners for inquiring into the State of Large Towns and Populous Districts . . . . . 333

2. A Bill for the Improvement of the Sewerage and Drainage of Towns and Populous Districts, and for making Provision for an ample supply of Water, and for otherwise promoting the Health and Convenience of the Inhabitants . . . . . . . ib.

ART. III.—Lectures on the more important Eruptive Fevers, Hemorrhages, and Dropsies, and on Gout and Rheumatism, delivered in the University of Pennsylvania. By N. CHAPMAN, M.D., Professor of the Theory and Practice of Medicine, &c. &c. . . . . . . 358

ART. IV.—Transactions of the Medical and Physical Society of Bombay for the Years 1842 and 1843 . . . . . . 373

ART. V.—Die Krankheiten des Gehirns und Ruckenmarks bei Kindern. Durch Krankheitsfälle aus dem ersten Kinderspitale erläutert, von Dr. L. W. MAUTHNER . . . . . . 381

A Treatise on the Diseases of the Brain and Spinal Chord in Children, illustrated by Cases occurring in the Wards of the First Children's Hospital. By Dr. W. L. MAUTHNER.

ART. VI.—Die angeborne Verrenkungen, mit Zwei Tafeln. Von LUDWIG JOSEPH MELICHER, Doctor der Medicin und Chirurgie, &c. &c. . . 393

Congenital Luxations. By LUDWIG J. MELICHER.

6 §

PAGE

ART. VII.—Mémoires de l'Académie Royale de Médecine . . . . 408

Memoirs of the Royal Academy of Medicine of Paris.

M. SÉGALAS on Uréthroplasty . . . . . . ib.

Dr. R. PRUS on Miningeal Apoplexy . . . 409

M. VALLEIX on Œdema of the Glottis . . . . 412

M. GINTRAC on Hereditary Transmission . . . . 417

Dr. C. BARON on Adventitious Products . . . . 418

Dr. BRIERRE DE BOISMONT on the Acute Delirium observed in Lunatic Asylums . . . . . . ib.

Dr. PAYAN on Extra-uterine Interstitial Pregnancy . . . 419

O. De LAFOND's Exposition of Experiments made upon Animals . 420

Professor HIPPOLITE LARREY's Essay on Penetrating Wounds of the Abdomen . . . . . . . 421

ART. VIII.—The Nature and Treatment of Cancer. By W. H. WALSHE, M.D., Professor of Pathological Anatomy in University College, Physician to University College Hospital, and to the Hospital for Consumption and Diseases of the Chest . . . . . . . 421

ART. IX.—Déontologie Médicale, ou des Devoirs et des Droits des Médecins dans l'Etat actuel de la Civilisation. Par le Docteur MAX. SIMON . . 438

Medical Deontology; or, the Duties and Rights of Medical Practitioners. By Dr. MAXIMILIAN SIMON.

ART. X.—The Descriptive and Physiological Anatomy of the Brain, Spinal Cord, and Ganglions, and of their Coverings. Adapted to the Use of Students. By ROBERT BENTLEY TODD, M.D., F.R.S., Professor of Physiology in King's College, London, &c. . . . . . . 452

ART. XI.—Vermischte Abhandlungen aus dem Gebiete der Heilkunde, von einer Gesellschaft practischer Aerzte zu St. Petersburg. Sechste Sammlung . 460

Medical Essays. By a Society of Physicians in St. Petersburg. Volume the Sixth.

Dr. HERZOG on Insanity . . . . . . ib.

Professor BUSCH, Cases of Sudden Death . . . 465

Dr. DOEPP's Report on the Foundling Hospital . . . 467

Dr. DOEPP's Operation for Varicose Aneurism . . . 470

Dr. WEISSE's Report on the Children's Hospital . . . 471

Dr. OCKEL on the Uses of Calendula and of Fuligo in the Diseases of Women 472

Dr. W. LERCHE's Report of the Private Ophthalmic Institution . 473

ART. XII.—An Essay on the Properties of Animal and Vegetable Life; their dependence on the Atmosphere, and Connexion with each other, in relation to the Functions of Health and Disease. By EDWARD JAMES SHEARMAN, M.D., &c. &c. . . . . . . . 473

ART. XIII.—Fauna Antiqua Sivalensis; being the Fossil Zoology of the Sewalik Hills, in the North of India. By HUGH FALCONER, M.D., F.R.S., F.L.S., F.G.S., &c. &c., and PROBY T. CAUTLEY, F.G.S., Captain in the Bengal Artillery, &c. &c. Part I. . . . . . . 476

PAGE

ART. XIV.—Mr. Churchill's Manuals . . . . . 479

A Manual of Physiology, including Physiological Anatomy; for the Use of the Medical Student. By WILLIAM B. CARPENTER, M.D., F.R.S., &c. . 480

A System of Practical Surgery. By WILLIAM FERGUSSON, Esq., F.R.S.E., Professor of Surgery in King's College, London, &c. Second Edition . 483

A Manual of Medical Jurisprudence. By ALFRED S. TAYLOR, F.R.S. Second Edition . . . , . . . 484

ART. XV.—The Practice of Surgery. By JAMES MILLER, F.R.S.E., Professor of Surgery in the University of Edinburgh, Surgeon to the Royal Infirmary, &c. &c. . . . . . . 488

ART. XVI.—Leçons sur les Maladies de la Peau, professées à l'Ecole de Médecine de Paris en 1841, 1842, 1843, 1844. Par ALPH. CAZENAVE, Médecin de l'Hôpital Saint Louis, &c. Livraisons I, II, III . . . 491

Lectures on Diseases of the Skin, delivered in the College of Medicine at Paris in 1841, 1842, 1843, 1844. By ALPH. CAZENAVE, Physician to the Hospital of St. Louis, &c. Parts I, II, III.

---

### PART SECOND.—Bibliographical Notices.

---

ART. I.—First Steps to Anatomy. By JAMES L. DRUMMOND, M.D., Professor of Anatomy and Physiology in the Royal Belfast Institution . . 493

ART. II.—Insect Life. By DAVID BADHAM, M.D., late Radcliffe Travelling Fellow of the University of Oxford, &c. . . . . 494

ART. III.—The Modern Treatment of Syphilitic Diseases, both Primary and Secondary. Comprising numerous formulæ for the preparation and mode of administration of the new remedies; and an account of a safe and successful mode of treating chronic, protracted, and constitutional syphilis, by the mercurial vapour-bath. By LANGSTON PARKER, Surgeon to the Queen's Hospital, Birmingham, &c. &c. Second Edition . . . 495

ART. IV.—A Natural History of the Mammalia. By G. R. WATERHOUSE, Esq., of the British Museum . . . . . 496

ART. V.—On Scarlatina and its Successful Treatment by the Acidum Aceticum Dilutum of the Pharmacopœia. By ISAAC B. BROWN . . 497

ART. VI.—On the Present State of Therapeutical Inquiry. By JAMES ARNOTT, M.D. 499

ART. VII.—Elements of Anatomy. By JONES QUAIN, M.D. Fifth Edition. Edited by RICHARD QUAIN and WILLIAM SHARPEY, M.D., Professors of Anatomy and Physiology, University College, London. Parts I, II . . 499

### PART THIRD.—Natural History and Treatment of Diseases.

PAGE

I.—Prefatory Notice. By the EDITOR . . . . . . 501

II.—On the Observation of Nature in the Treatment of Disease. By ANDREW COMBE, M.D., Fellow of the Royal College of Physicians of Edinburgh, one of the Physicians in Ordinary, in Scotland, to the Queen, &c. &c. In a Letter to the Editor . . . . . . . 505

III.—On the Methods for obtaining a Natural History of Diseases. By THOMAS LAYCOCK, M.D. In a Letter to the Editor . . . 525

IV.—On the Patronage of Quacks and Impostors by the Upper Classes of Society. By the EDITOR . . . . . . 533

### PART FOURTH.—Original Reports and Memoirs.

Report on the Progress of Human Anatomy and Physiology in the years 1844-5. By JAMES PAGET, Lecturer on General and Morbid Anatomy and Physiology, and Warden of the Collegiate Establishment, at St. Bartholomew's Hospital . . . . . . 541

Books received for Review . . . . . . 570

INDEX, TITLE, &C.

